# Staphylococcus lugdunensis: Review of Epidemiology, Complications, and Treatment

**DOI:** 10.7759/cureus.8801

**Published:** 2020-06-24

**Authors:** Shridhar Parthasarathy, Shrey Shah, Avinaash Raja Sager, Anvitha Rangan, Satya Durugu

**Affiliations:** 1 Biology, The College of New Jersey, Ewing, USA; 2 Internal Medicine, Jawaharlal Nehru Medical College, Belgaum, IND; 3 Internal Medicine, University of Louisville, Louisville, USA

**Keywords:** staphylococcus lugdunensis, coagulase-negative staphylococci, infective endocarditis, bone infection, prosthetic joint infection, skin and soft tissue infection, pet

## Abstract

*Staphylococcus lugdunensis *is a species of coagulase-negative staphylococci (CNS) that induces a variety of infectious diseases, including skin and soft tissue infection (SSTI), infective endocarditis (IE), and bone and PJI. This review article underscores the important points in the literature about *S. lugdunensis *infections, including its epidemiology, diagnosis, and treatment, as well as specific types of infections it can cause. Anatomical and age-related distributions of *S. lugdunensis *SSTIs have been noted, though they most commonly occur as abscesses. *S. lugdunensis *can also manifest as an aggressive form of IE presenting with valve destruction and abscess formation, frequently requiring surgery and with a high mortality rate. Bone and joint infections caused by *S. lugdunensis *are also more invasive than infections by other species of CNS. The clinical presentation of *S. lugdunensis *infection in SSTI, IE, and bone/joint infection is frequently more similar to that of *S. aureus *infection than that of other CNS infections, necessitating species-level differentiation of CNS for proper diagnosis. Though historically, this depended upon biochemical tests that were neither routine nor reliable, the implementation of matrix-assisted laser desorption/ionization time of flight mass spectrometry (MALDI-TOF MS) in clinical laboratories has made identification of CNS species such as *S. lugdunensis *more practical. Imaging modalities, especially the fluorodeoxyglucose (FDG) with positron emission tomography (PET), are another important emerging trend in the diagnosis of infectious diseases such as *S. lugdunensis *infection. *S. lugdunensis *remains highly susceptible to a wide gamut of antibacterial therapies, which is uncharacteristic of other CNS. Infections can usually be treated by antibiotics traditionally used for CNS such as oxacillin. The breakpoints for *S. lugdunensis *are higher than those of other CNS and similar to *S. aureus* breakpoints. In the case of aggressive IE or bone/joint infection by *S. lugdunensis*, it is recommended to treat with a β-lactam agent. Further study is needed to understand the diversity, virulence, and population structure of this species, as well as its role in other infections, such as urinary tract infections (UTIs), respiratory infections, peritonitis, and bacteremia.

## Introduction and background

*Staphylococcus lugdunensis *is a species of coagulase-negative staphylococci (CNS), known to be a normal skin commensal that preferentially colonizes the perineal region [1-3]. In recent years, a growing body of evidence demonstrates the role of *S. lugdunensis *in a wide spectrum of diseases. Though the most common of these are skin and soft tissue infections (SSTIs), *S. lugdunensis *has also been shown to infrequently cause bone, joint space, and prosthetic joint infections (PJIs); aggressive valvular endocarditis with significant mortality rates; urinary tract infections; and peritonitis [1,4-15]. These infections may differ greatly from other CNS infections in clinical presentation and treatment options; hence, an understanding of specific features of *S. lugdunensis* infection has particular clinical value. 

This review article aims to highlight salient points pertaining to *S. lugdunensis* within the existing literature, particularly relating to its pathogenesis, microbiology, treatment modalities, and emerging trends.

## Review

Epidemiology

*S. lugdunensis *physiological colonization is estimated to be present in 30% to 50% of patients, mostly in the inguinal area with a substantial presence in the axilla and nares as well [16]. It has a low presence in human clinical samples. A review of studies between 2001 and 2011 in human clinical samples found *S. lugdunensis *presence ranging from 0.5% to 9% in CNS-positive samples, while one study in 2015 also identified 3.6% of CNS-positive blood cultures to be *S. lugdunensis *[17,18]. Other studies have consistently shown *S. lugdunensis* to represent under 3% of CNS in human samples [19]. The incidence of *S. lugdunensis *SSTIs is estimated to be 53 per 100,000 per year [1]. *S. lugdunensis *bacteremia (SLB) incidence has been estimated at 5.6 patients per 100,000 admissions, while the incidence of clinically relevant SLB was 1.3 patients per 100,000 admissions [20].

*S. lugdunensis *is nonetheless an important pathogen. It has been reported as the second most common pathogen, behind *S. epidermidis*, in infective endocarditis (IE) resulting from CNS [21]. In a study in which other CNS species were microbiologically relevant in under 26% of cases, *S. lugdunensis *had 40% microbiological relevance [22]. In another, seven of eight identified cases of SLB were determined to be clinically significant [18]. 

Skin and soft tissue infection

SSTIs account for the majority of *S. lugdunensis *infections, with Bocher et al., as described above, having identified an incidence of 53 per 100,000 per year, representing an increase from earlier pilot studies [1,4,23]. Though less common than SSTIs caused by other CNS and *S. aureus*, it produces a more virulent clinical picture in contrast to the CNS and more closely resembling that of *S. aureus *[1].

These infections commonly affect the middle-aged to elderly patient populations, with greater prevalence in females [1,5]. Approximately half of all patients affected have some form of concurrent comorbidity, either in the form of chronic immunosuppressive therapy, diabetes mellitus, or a history of trauma to the site [4,5]. Anatomically, previous studies have shown *S. lugdunensis *infections to be distributed predominantly below the pelvic girdle or in the inguinal area, though Herchline and Ayers noted a broader spectrum of localization [4,24,25]. This is supported and built on by Bocher et al., who posited an age-related distribution of SSTI sites: among children, otitis externa is most common; among the middle-aged, apocrine gland-related areas such as the axilla, buttocks, groin and mammary regions; and among the elderly, infections and ulcers of the digits [1]. 

Abscesses are the most common manifestation of *S. lugdunensis *infections [1]. In a case series of five patients with cutaneous *S. lugdunensis *infection, Heldt Manica and Cohen also noted cellulitis, cyst formation, or pustules as a mode of presentation. All five patients presented without any other constitutional symptoms. Almost all patients require incision and drainage along with antibiotic therapy; and the rare few requiring only monotherapy are those with superficial infections [1,5]. *S. lugdunensis *demonstrated an antibiotic sensitivity similar to methicillin-sensitive *S. aureus*, and some patients may require prolonged therapy [5].

Infective endocarditis

Infrequently, *S. lugdunensis *manifests as an aggressive form of IE presenting with valve destruction and abscess formation. In a literature review ranging from 1988 to 2008 with a total of 67 documented cases, Liu et al. found that *S. lugdunensis *IE predominantly affected the left side of the heart and formed vegetations demonstrable on echocardiography [9]. These infections were more prevalent in males, and the majority of infected patients were middle-aged with a history of comorbidity [9].

In contrast to other CNS infections, which tend to be acquired at the hospital and to affect prosthetic valves and indwelling devices, *S. lugdunensis *mainly affects native heart valves and is more likely to be acquired through the community without an identifiable source of infection [9,26]. The presentation of *S. lugdunensis *IE has been noted to more closely resemble that of *S. aureus *IE compared to IE due to other CNS species [9].

Antibiotic therapy alone is usually not sufficient and patients often require surgical interventions with valve replacement. The need for surgery is much higher than that of *S. aureus *IE (70% vs. 37%) and comparable to that of *S. epidermidis *IE - though with significantly higher mortality rates [9,27]. Surgery is the only independent risk factor for mortality [9]. Unlike other CNS, most cases of *S. lugdunensis *are susceptible to a wide array of antimicrobials especially penicillins, though conservative management alone is often never satisfactory [9]. The Infectious Diseases Society of America guidelines for endocarditis due to *S. lugdunensis* recommends β-lactam agent treatment along with monitoring for the development of either periannular extension or extracardiac spread of infection [28]. 

Native bone, prosthetic joint and vertebral space infections

Another rising trend is the involvement of *S. lugdunensis *in orthopedic diseases. *S. lugdunensis *mostly affects prosthetic joints causing PJIs, and an equal amount of native joint and spondylodiscitis/vertebral osteomyelitis. These infections are more prevalent in males, and typically manifest in middle age [7]. In PJIs, the knee joint is more frequently affected than the hip joint [6].

In a study of the largest cohort of its kind, Shah et al. found that PJIs due to *S. lugdunensis *tend to be more invasive than other CNS species and are more similar to those due to *S. aureus*: infections presented with an acute clinical picture of copious purulent discharge and rapid tissue destruction [6]. A majority of patients have underlying comorbidities or a history of immunosuppression, including those due to diabetes mellitus, chronic steroid therapy, or, most commonly, some form of urogenital anomaly [6,7]. This relation to underlying urogenital anomalies is well documented in other literature as well, though its role cannot be established without case-control studies [3,4,28,29]. The majority of patients with bone/joint space infections and PJI require surgical intervention along with parenteral antibiotic therapy [6,7]. A noteworthy exception is in the case of spondylodiscitis and vertebral osteomyelitis, where successful resolution may be achieved without surgery [7].

Based on current Clinical Laboratory Standards Institute guidelines, an *S. aureus *interpretative breakpoint for oxacillin should be used, meaning a MIC of 1 microgram/ml is deemed oxacillin sensitive for *S. lugdunensis *but resistant for other CNS [6,23]. β-lactams are preferred over vancomycin due to their more rapid bactericidal action and better penetration into bone, and display fewer adverse effects, such as hypotension and red man syndrome, compared to vancomycin [30-32]. Patients treated with parenteral β-lactams are also more likely to be free of treatment failure at a two-year interval than those who received parenteral vancomycin [6].

Diagnosis

Diagnosing a CNS such as *S. lugdunensis *begins with a clinical presentation suspicious for a staphylococcus infection, which is frequently expected to be a coagulase-positive staphylococcus infection (e.g., *S. aureus*). A bacterial culture is then performed in which CNS can be detected; however, CNS detection alone may not be sufficient to determine clinical significance. This difficulty arises from the possibility that a CNS-positive culture may result from contamination of the specimen or colonization of skin or mucous membranes rather than a clinically relevant infection [17]. For example, *S. lugdunensis *is also commonly isolated as a part of a mixed flora [14]. Often, a second positive culture result is also obtained before a CNS infection is diagnosed [33].

Historically, identifying individual CNS species has not been common in clinical laboratories. Distinguishing CNS from *S. aureus*, however, has been traditionally achieved by assays based on staphylococcal coagulase, or clumping factor. In particular, the clumping factor has been detected by the slide coagulase test or latex agglutination test, both of which detect both free and membrane-bound forms of coagulase. *S. lugdunensis*, which lacks free coagulase but, in up to 65% of isolates, possesses a membrane-bound form, is often mistaken for *S. aureus *following such tests as a result [34]. The tube coagulation test, which is more specific to free coagulase, is a traditional and more reliable method for CNS detection, but it is limited by long incubation time. The “third-generation,” rapid latex, and hemagglutination assays that have been developed to circumvent these constraints are, however, themselves limited by lower specificity for *S. aureus *identification, likely due to clumping factor-positive CNS species including *S. lugdunensis *[17].

Species-level identification of *S. lugdunensis *depends on its specific diagnostic characteristics. This species forms cream-white to slightly yellow colonies is oxidase-negative and is not novobiocin-resistant [17]. Cultures commonly produce a ‘hypochlorite bleach odor’ similar to that of *Eikenella corrodens *in Colombia agar [1]. Additionally, Kleiner et al. and Liu et al. have noted the ability of *S. lugdunensis *to form biofilm and a glycocalyx coat, respectively, lending to its virulence [9,23,35]. Additionally, *S. lugdunensis *is positive for ornithine decarboxylase activity and pyrrolidonyl arylamidase activity in over 90% of isolates, and this test can yield results within eight hours [19]. Additionally, nucleic acid-based assays, including real-time polymerase chain reaction (PCR) for specific conserved genes such as the 16S or 23S ribosomal DNA, have a higher identification rate for *S. lugdunensis *[16]. These tests have not been established as routine procedures, but are especially useful in the event of inconclusive or unclear results from other procedures [17].

Over the past decade, the increasing implementation of matrix-assisted laser desorption/ionization time of flight mass spectrometry (MALDI-TOF MS) in laboratories has resulted in simpler, faster, more cost-effective, and increasingly accurate *S. lugdunensis *identification. This proteomic method relies on manufacturers’ databases whose spectra content is growing, resulting in nearly 100% sensitivity and specificity [16]. Furthermore, MALDI-TOF MS can reliably identify *S. lugdunensis *directly from blood cultures [16]. The benefits of this test include fewer preparatory steps, as well as significantly reduced time since identification can be achieved within one hour [36]. Implementation of MALDI-TOF MS has resulted in drastic increases in *S. lugdunensis *detection in multiple studies. In one case, routine use of this technique coincided with a notable uptick in diagnosed *S. lugdunensis *urinary tract infections [37]. In another, there was an 18-fold increase in *S. lugdunensis *identification in the two years following MALDI-TOF MS implementation compared to the preceding two years [22]. Evidently, MALDI-TOF MS has established itself as a prime tool for identifying CNS at the species level and is therefore invaluable for the diagnosis of *S. lugdunensis *infection. 

In recent years, imaging modalities have also shown to be effective in diagnosing infections, namely through the combined use of fluorodeoxyglucose (FDG) with positron emission tomography (PET) [38,39]. There has been an increasing trend to utilize FDG-PET in non-oncological fields such as infectious disease, cardiology, neurology, and gastroenterology [40-43]. FDG is a tracer which, like glucose, is uptaken in greater quantities by cells displaying higher metabolic activity. Coupling FDG with PET imaging, which has greater spatial resolution than alternative techniques, yields valuable molecular-level information for a variety of disorders [38]. Specifically, by localizing in regions of increased metabolic activity, FDG can delineate abnormalities in the body, including inflammatory response to infectious diseases, and is therefore an important diagnostic tool for conditions such as *S. lugdunensis *infection. 

Treatment

In the treatment of CNS such as *S. lugdunensis*, the first line of antibacterial treatment is isoxazolyl penicillin, such as oxacillin. Fortunately, *S. lugdunensis *remains highly susceptible to a wide gamut of antibacterial therapies, which is uncharacteristic of other CNS such as *S. epidermidis *[34]. However, previous literature indicates the development of resistance to streptomycin, erythromycin, ceftazidime, and gentamicin through isolated case reports [16,34]. Also, Kragsbjerg et al. describe in a case report the development of resistance in *S. lugdunensis *to rifampicin and ciprofloxacin while treating a chronic *S. lugdunensis *infection [44]. However, successful treatment of *S. lugdunensis *with linezolid after the failure of ciprofloxacin and rifampin have been reported in a patient with a PJI [45]. Furthermore, *S. lugdunensis *resistance to penicillin has reported being considerably high at 45% in the United States [16]. The Clinical Laboratory and Standards Institute (CLSI) has recommended that *S. lugdunensis *isolates should be screened for the penicillin-binding protein 2A by latex agglutination or the mecA gene by PCR, both of which confer resistance to oxacillin and other β-lactams [46]. Additionally, CLSI guidelines also state breakpoints for *S. lugdunensis *that are higher than those of most other CNS [46]. Additionally, *S. lugdunensis *and *S. aureus *have the same breakpoints: susceptible isolates have MICs of ≤2 µg/ml, while resistant isolates have MICs ≥4 µg/ml. Vancomycin resistance in *S. lugdunensis *has not been documented to this date [46].

SSTIs of *S. lugdunensis *are indistinguishable visually from *S. aureus*. However, compared to other CNS, *S. lugdunensis *is associated with a higher complication rate [1]. Anguera et al. observed in a prospective cohort of ten patients with IE caused by *S. lugdunensis *that there was no difference in mortality between antibacterial monotherapy and antibacterial combination therapy [47]. However, there exists a lack of data in the literature on the efficacy of antibacterial treatments for *S. lugdunensis *infections currently. In terms of treatment, a vast majority of patients with a *S. lugdunensis *infection were treated primarily with surgical incision or antibiotics, while most other patients had superficial wound infections [1].

*S. lugdunensis*, like other CNS, is able to produce a biofilm that makes infections become significantly more difficult to treat even with the high susceptibility of *S. lugdunensis *to most antibiotic treatments [1]. Unfortunately, little is known about the diversity, virulence, and population structure of *S. lugdunensis*. Even though *S. lugdunensis* is highly susceptible to a myriad of antibiotic therapies,* S. lugdunensis *is more aggressive than other CNS as *S. lugdunensis *carries a high mortality rate in endocarditis patients [16]. Thus, if *S. lugdunensis *is isolated, transesophageal echocardiography should be conducted to evaluate for IE [48-50]. Treatment with a β-lactam and debridement of foreign material should be pursued [48-50].

## Conclusions

*S. lugdunensis *is not an uncommon cause of infection. Its occurrence warrants our further attention due to its significant virulence and a broad range of pathology. Previous literature highlights some of the important aspects of this organism - particularly its predilection for SSTIs, aggressive clinical course, a possible relationship with immunosuppression/comorbidity, and a wide spectrum of antibiotic susceptibilities. The significant differences in presentation and management of infections by *S. lugdunensis *compared to other staphylococcal infections necessitate species-level differentiation of these organisms, a process which has recently become much more efficient, reliable, and routine due to the implementation of MALDI-TOF MS in clinical laboratories. Furthermore, though possessing a wide array of antibiotic susceptibilities due to low genetic diversity, *S. lugdunensis *has an evolving resistance that must be highlighted.

Future studies should search and identify biases and shortcomings of current literature on *S. lugdunensis *and improve on them, in the hope of consolidating the clinical correlations mentioned in this article. Lastly, more studies are required to shed some light on the role of this unique organism in causing UTIs, respiratory infections, peritonitis, and bacteremia.
